# Supplementary material for: Yes-Associated Protein Is Required for ZO-1-Mediated Tight-Junction Integrity and Cell Migration in E-Cadherin-Restored AGS Gastric Cancer Cells
Source: Biomedicines. 2021 Sep 18;9(9):1264. doi: 10.3390/biomedicines9091264 (PMC8467433; doi:10.3390/biomedicines9091264)
Supplement: Supplementary file 1 [file biomedicines-09-01264-s001.zip › Fig. S1.pdf]

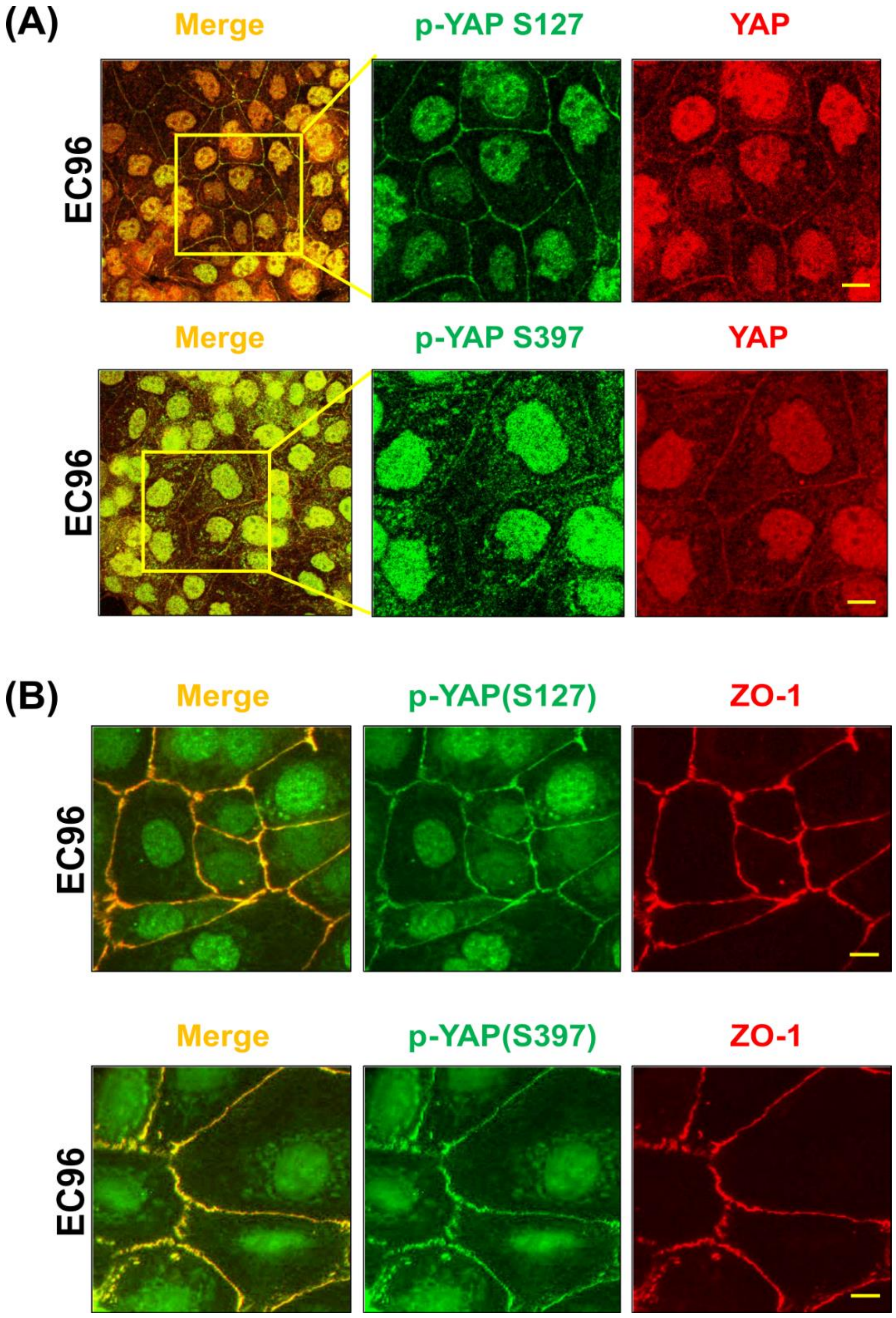

Fig. S1. Phosphorylated YAP localizes at cell membrane. EC96 cells were subjected to IF analyses using anti-p-YAP S127 or anti-p-YAP S397 antibodies, together with an anti-YAP antibody (A) or an anti-ZO-1 antibody (B). Scale bar = 10 μm.
