# Supplementary material for: Yes-Associated Protein Is Required for ZO-1-Mediated Tight-Junction Integrity and Cell Migration in E-Cadherin-Restored AGS Gastric Cancer Cells
Source: Biomedicines. 2021 Sep 18;9(9):1264. doi: 10.3390/biomedicines9091264 (PMC8467433; doi:10.3390/biomedicines9091264)
Supplement: Supplementary file 1 [file biomedicines-09-01264-s001.zip › Fig. S2.pdf]

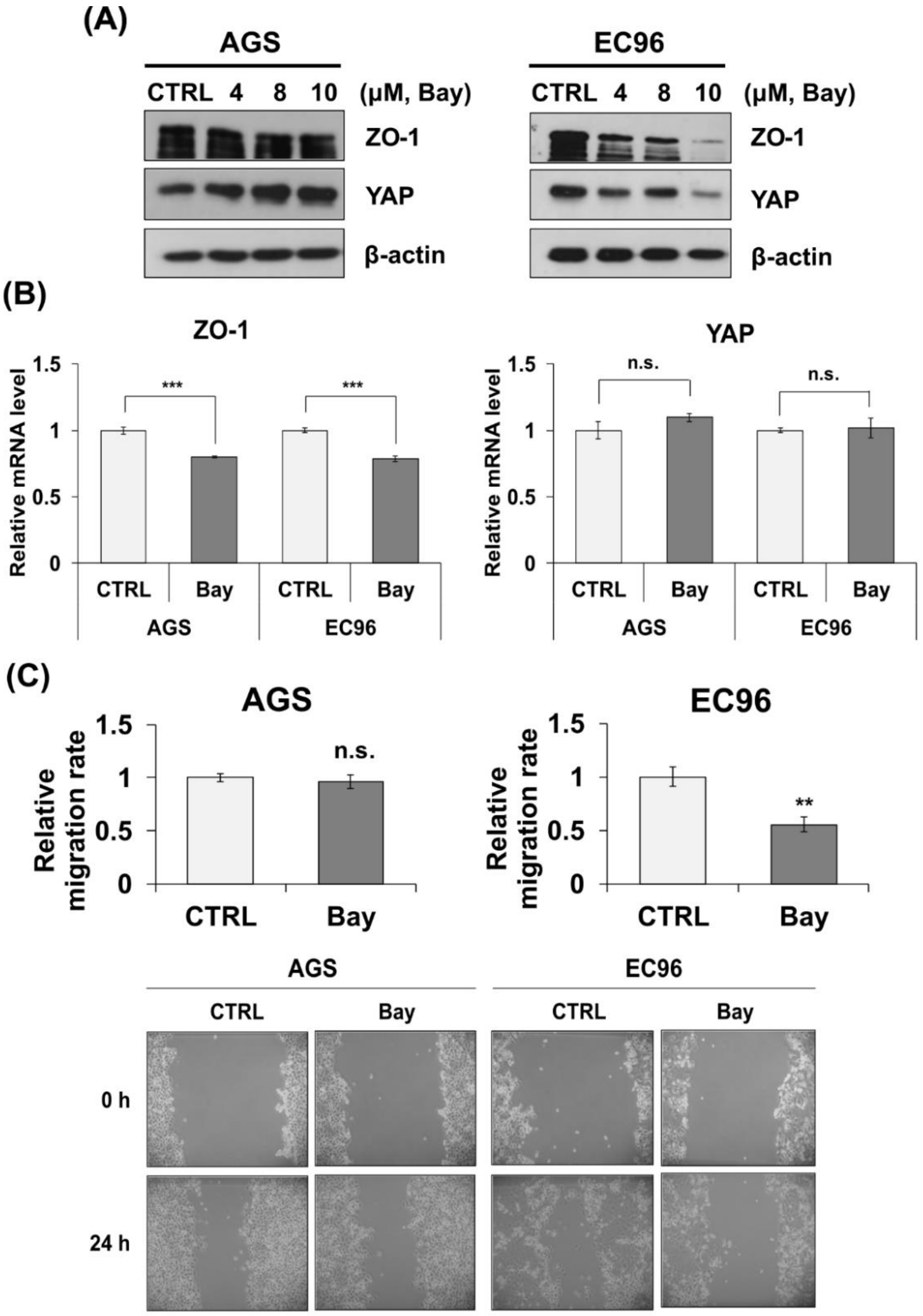


**Fig. S2. NF-κB signaling regulates ZO-1 expression and cell migration.** AGS and EC96 cells were treated with Bay 11-7082 (Bay) at the indicated concentrations and subjected to immunoblot analysis using the indicated antibodies (A), qRT-PCR analysis (B) or cell migration assay (C). n.s. = not significant, **P < 0.01 and ***P < 0.001.
