# Supplementary material for: Yes-Associated Protein Is Required for ZO-1-Mediated Tight-Junction Integrity and Cell Migration in E-Cadherin-Restored AGS Gastric Cancer Cells
Source: Biomedicines. 2021 Sep 18;9(9):1264. doi: 10.3390/biomedicines9091264 (PMC8467433; doi:10.3390/biomedicines9091264)
Supplement: Supplementary file 1 [file biomedicines-09-01264-s001.zip › Fig. S3.pdf]

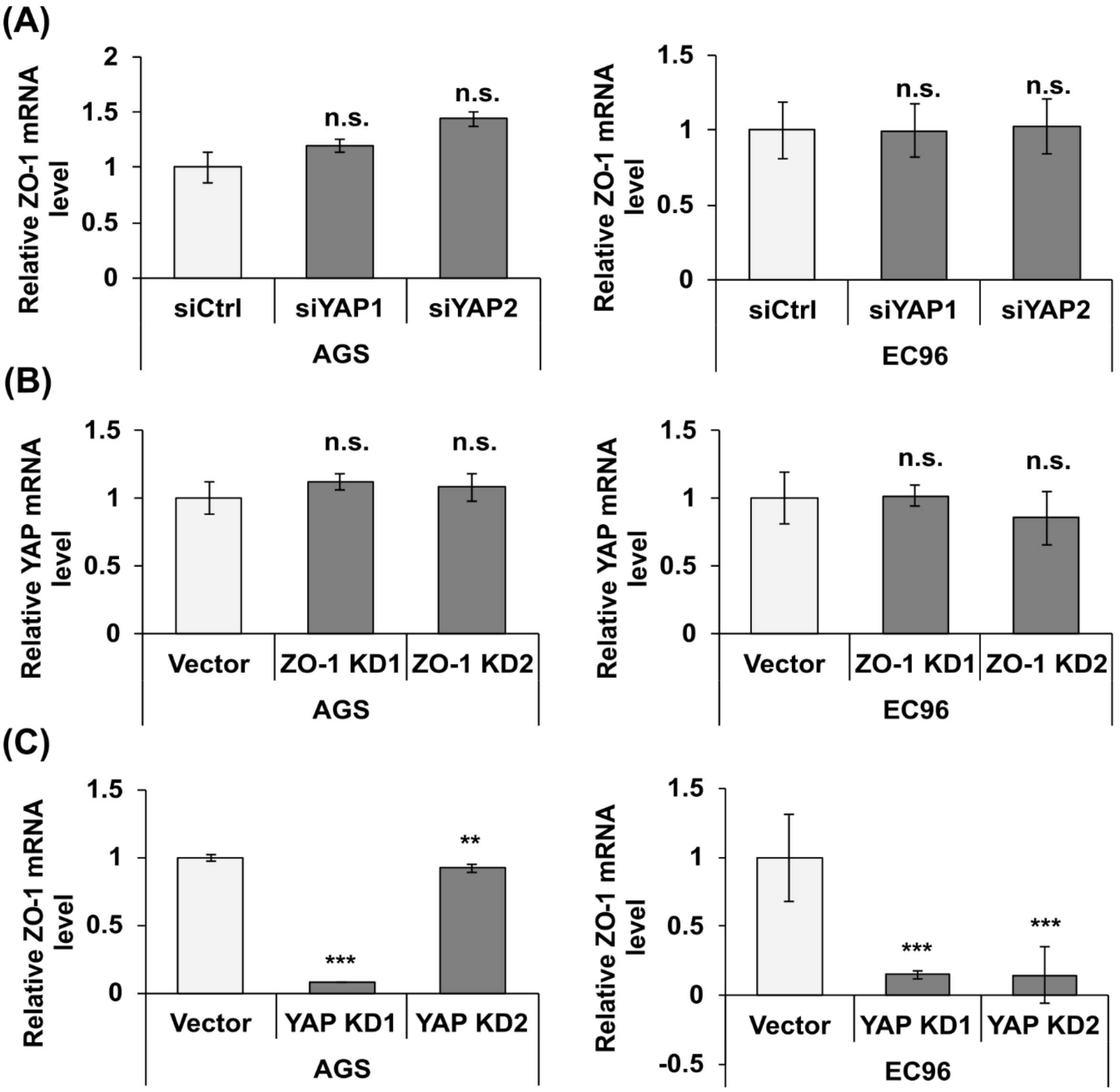


**Fig. S3. YAP and ZO-1 expression is regulated reciprocally.** Cells transfected with siYAP RNA (A), ZO-1 KD cells (B), or YAP KD cells (C) were subjected to qRT-PCR analysis. n.s. = not significant, **P < 0.01 and ***P < 0.001.
