# Supplementary material for: Yes-Associated Protein Is Required for ZO-1-Mediated Tight-Junction Integrity and Cell Migration in E-Cadherin-Restored AGS Gastric Cancer Cells
Source: Biomedicines. 2021 Sep 18;9(9):1264. doi: 10.3390/biomedicines9091264 (PMC8467433; doi:10.3390/biomedicines9091264)
Supplement: Supplementary file 1 [file biomedicines-09-01264-s001.zip › Fig. S4.pdf]

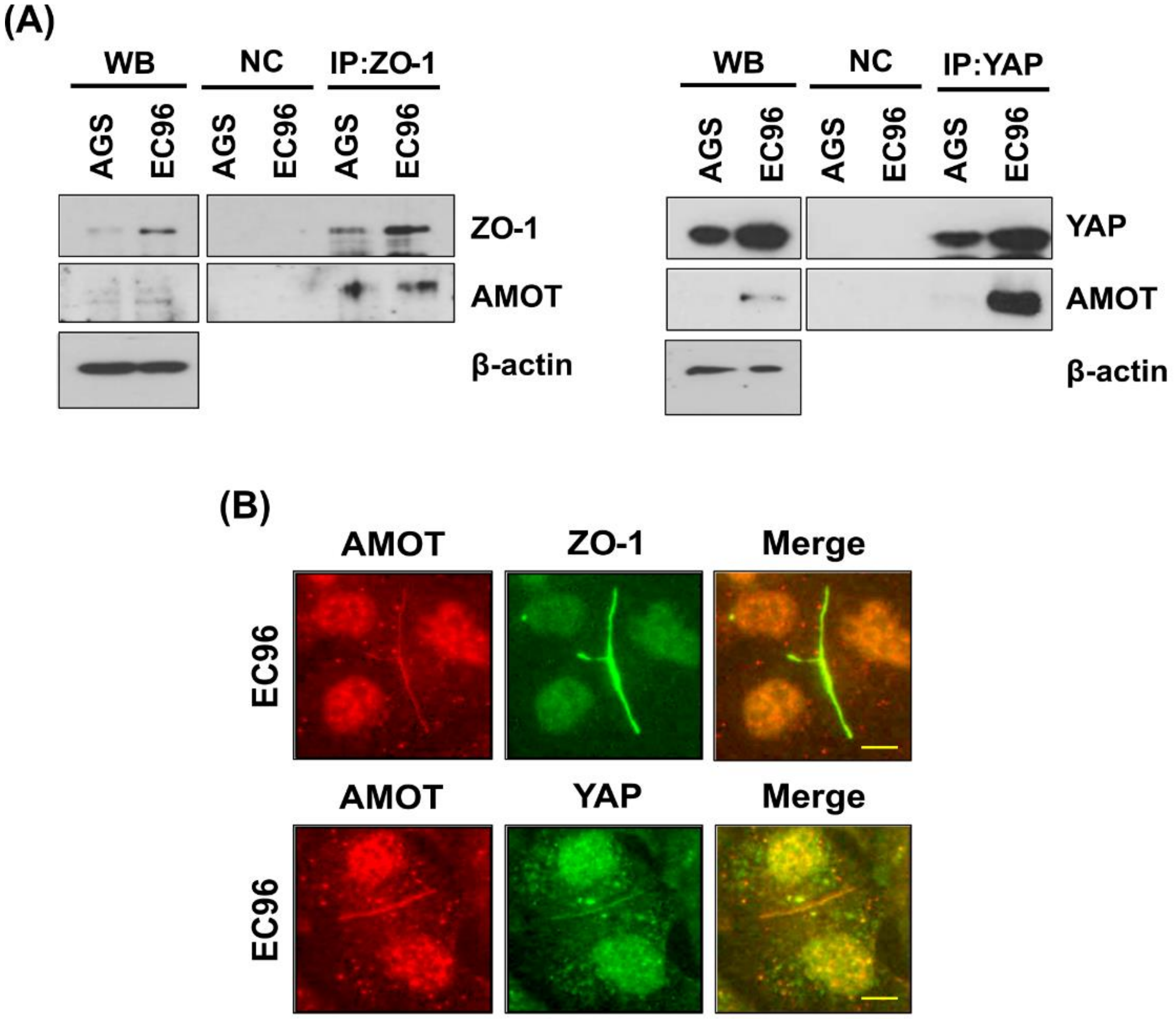

Fig. S4. AMOT is associated with YAP and ZO-1. (A) AGS and EC96 cells were subjected to IP analysis using anti-ZO-1 and anti-YAP antibodies and precipitates were subjected to immunoblot analysis for AMOT. (B) AGS and EC96 cells were subjected to IF analysis of AMOT to evaluate co-localization with ZO-1 or YAP. Scale bar = 10 μm.
