# Supplementary material for: Yes-Associated Protein Is Required for ZO-1-Mediated Tight-Junction Integrity and Cell Migration in E-Cadherin-Restored AGS Gastric Cancer Cells
Source: Biomedicines. 2021 Sep 18;9(9):1264. doi: 10.3390/biomedicines9091264 (PMC8467433; doi:10.3390/biomedicines9091264)
Supplement: Supplementary file 1 [file biomedicines-09-01264-s001.zip › Fig. S5.pdf]

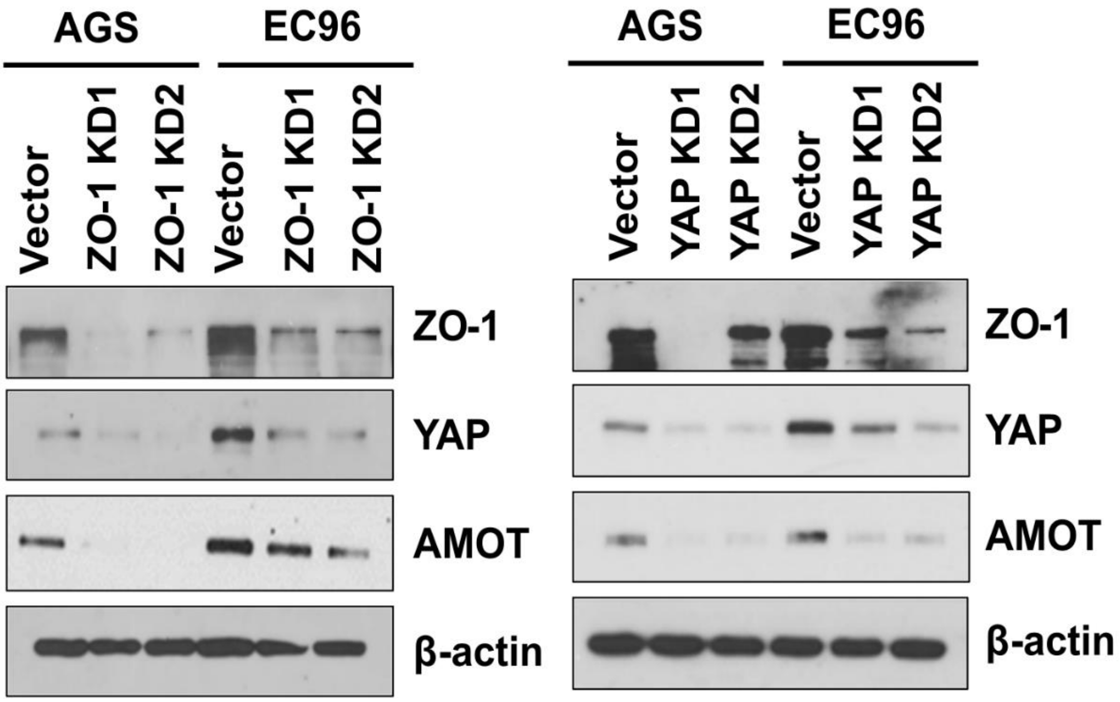

Fig. S5. Regulation of AMOT expression in ZO-1 KD or YAP KD cells. AGS and EC96 ZO-1 KD cells (left panel) or YAP KD cells (right panel) were subjected to immunoblot analysis using the indicated antibodies.
