# Supplementary material for: Yes-Associated Protein Is Required for ZO-1-Mediated Tight-Junction Integrity and Cell Migration in E-Cadherin-Restored AGS Gastric Cancer Cells
Source: Biomedicines. 2021 Sep 18;9(9):1264. doi: 10.3390/biomedicines9091264 (PMC8467433; doi:10.3390/biomedicines9091264)
Supplement: Supplementary file 1 [file biomedicines-09-01264-s001.zip › Fig. S6.pdf]

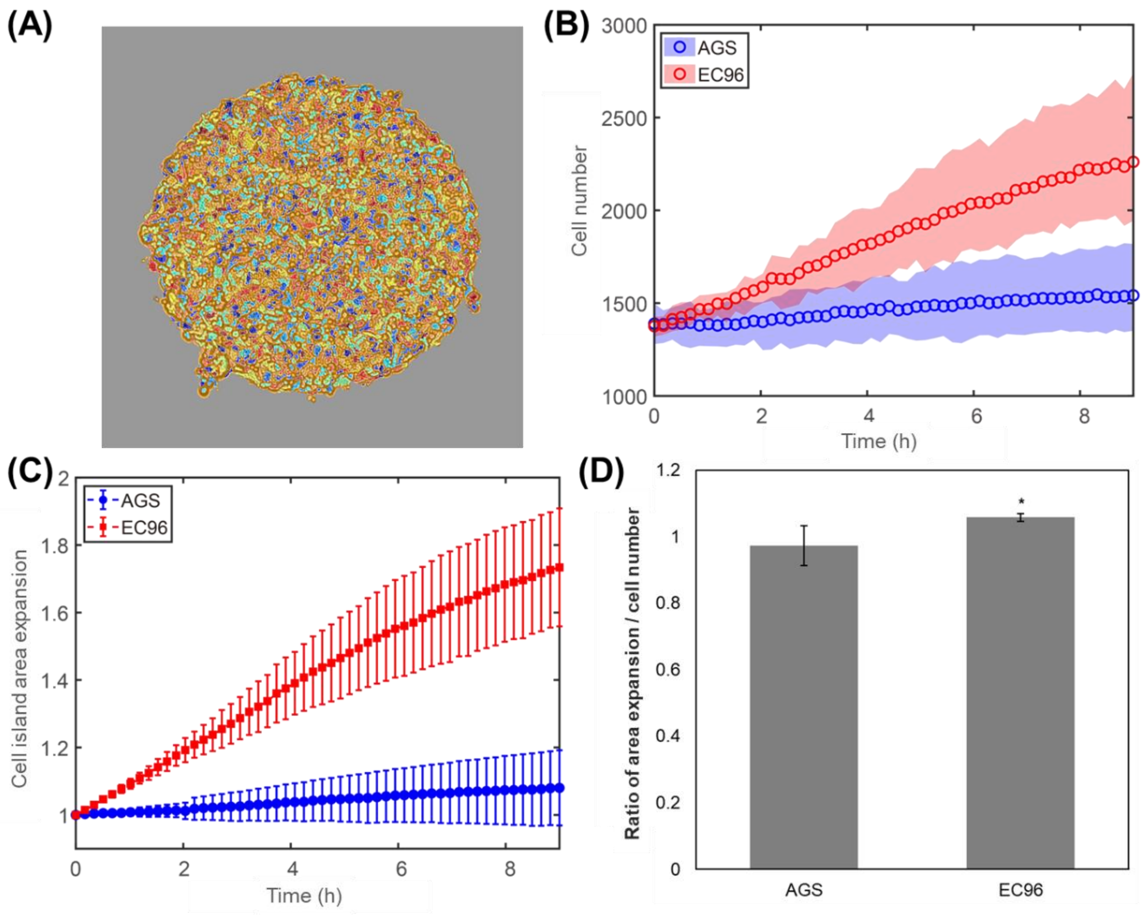

Fig. S6. Analysis of relationship between cell proliferation and cell island area expansion in AGS and EC96. (A) Each cell color label indicated the individual cells that were counted in a cell island. (B) Cell number was counted and plotted according to elapsed time after initial measurement to show increase of cell number in a time-dependent manner. Averages of five independent experiments are presented by circle and error bars are presented by color area. (C) Cell island area expansion was measured according to elapsed time. Averages of five independent experiments with error bars are presented. (D) Expanded cell island was divided by initial cell number and the ratio was presented. The error bars represent the standard errors calculated from separate five assays on each group. * $p < 0.05$
