# Supplementary material for: Yes-Associated Protein Is Required for ZO-1-Mediated Tight-Junction Integrity and Cell Migration in E-Cadherin-Restored AGS Gastric Cancer Cells
Source: Biomedicines. 2021 Sep 18;9(9):1264. doi: 10.3390/biomedicines9091264 (PMC8467433; doi:10.3390/biomedicines9091264)
Supplement: Supplementary file 1 [file biomedicines-09-01264-s001.zip › Supplementary Figure.pdf]

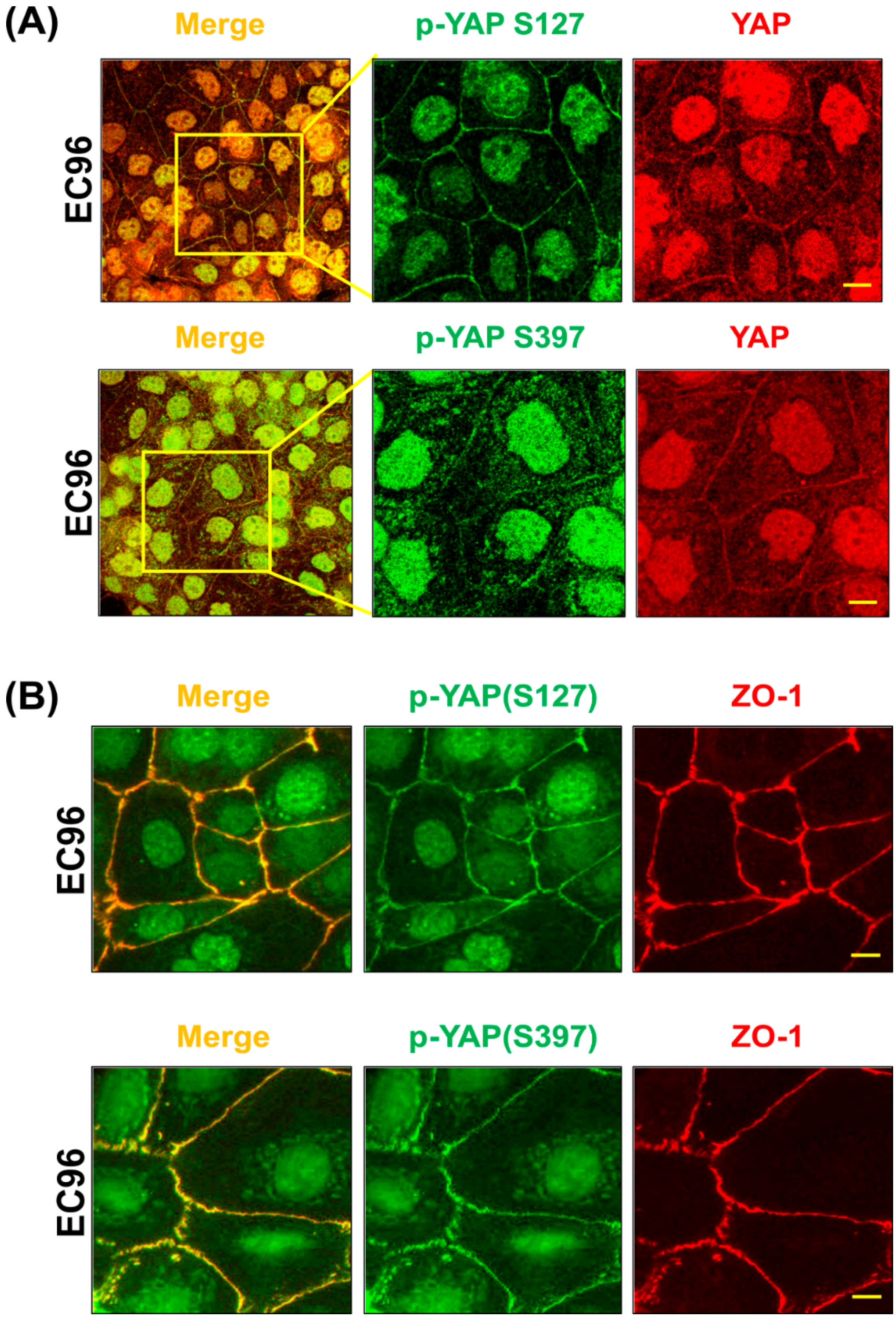

Fig. S1. Phosphorylated YAP localizes at cell membrane. EC96 cells were subjected to IF analyses using anti-p-YAP S127 or anti-p-YAP S397 antibodies, together with an anti-YAP antibody (A) or an anti-ZO-1 antibody (B). Scale bar = 10 μm.

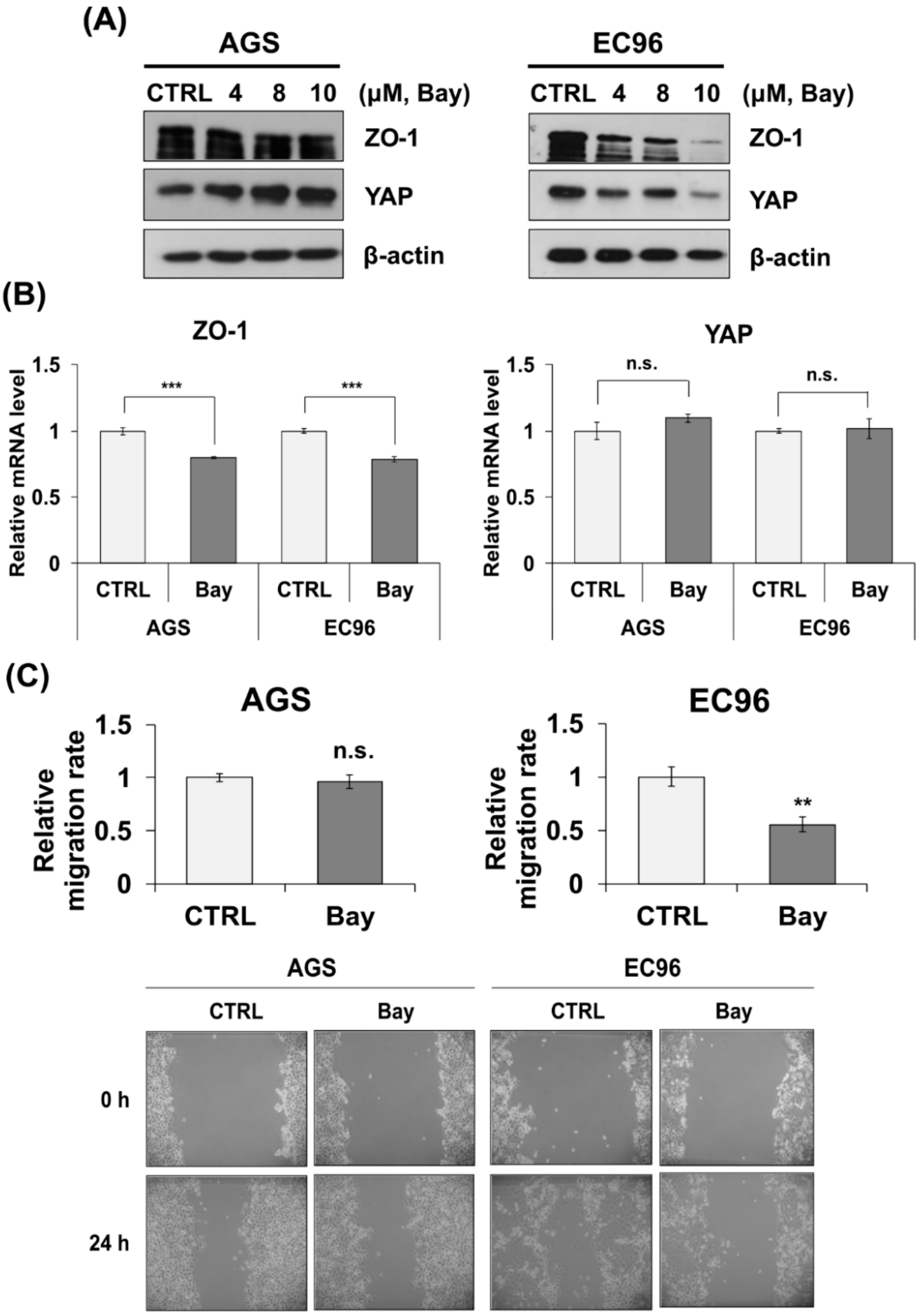

Fig. S2. NF-κB signaling regulates ZO-1 expression and cell migration. AGS and EC96 cells were treated with Bay 11-7082 (Bay) at the indicated concentrations and subjected to immunoblot analysis using the indicated antibodies (A), qRT-PCR analysis (B) or cell migration assay (C). n.s. = not significant, **P < 0.01 and ***P < 0.001.

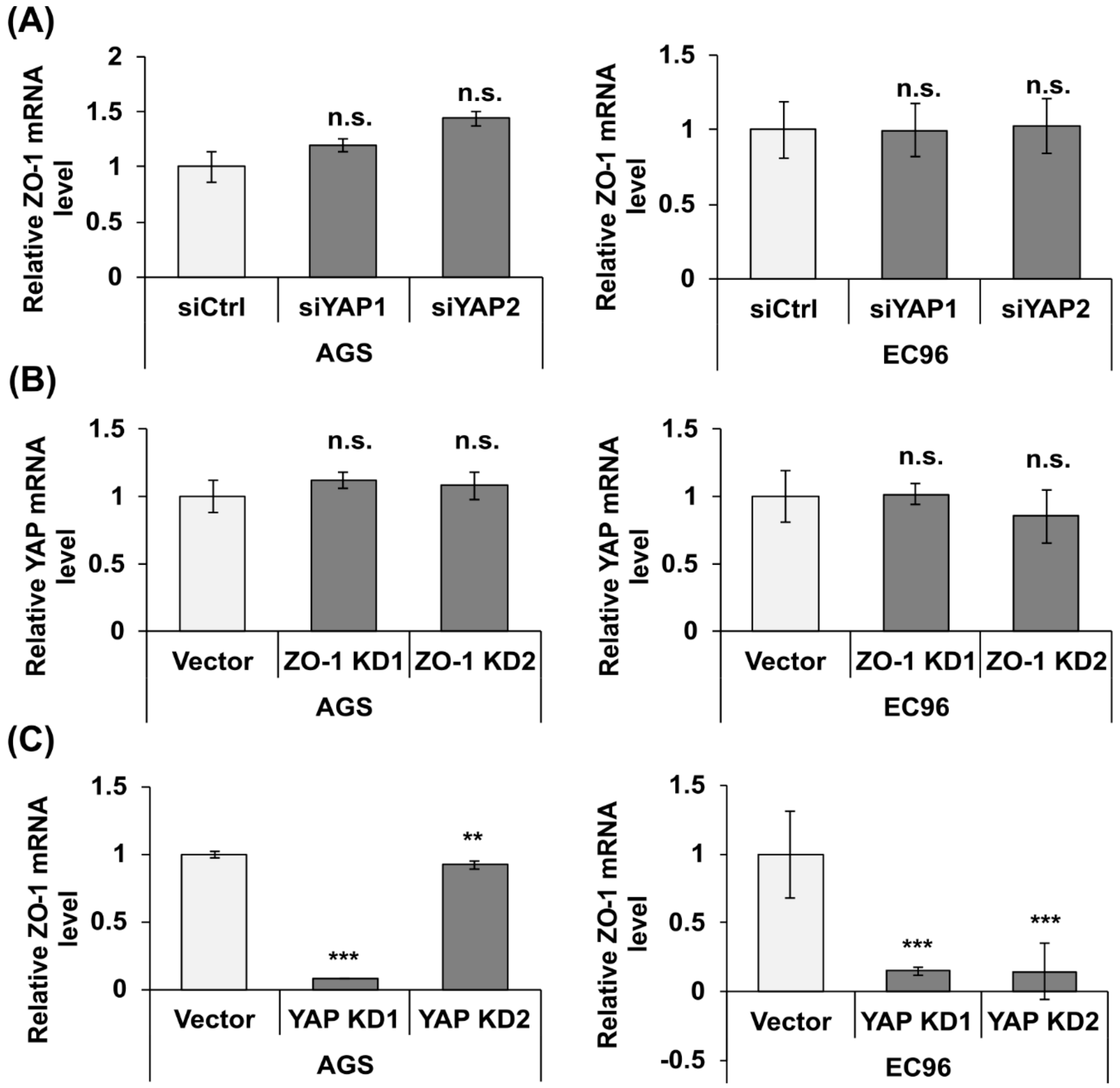

Fig. S3. YAP and ZO-1 expression is regulated reciprocally. Cells transfected with siYAP RNA (A), ZO-1 KD cells (B), or YAP KD cells (C) were subjected to qRT-PCR analysis. n.s. = not significant, **P < 0.01 and ***P < 0.001.

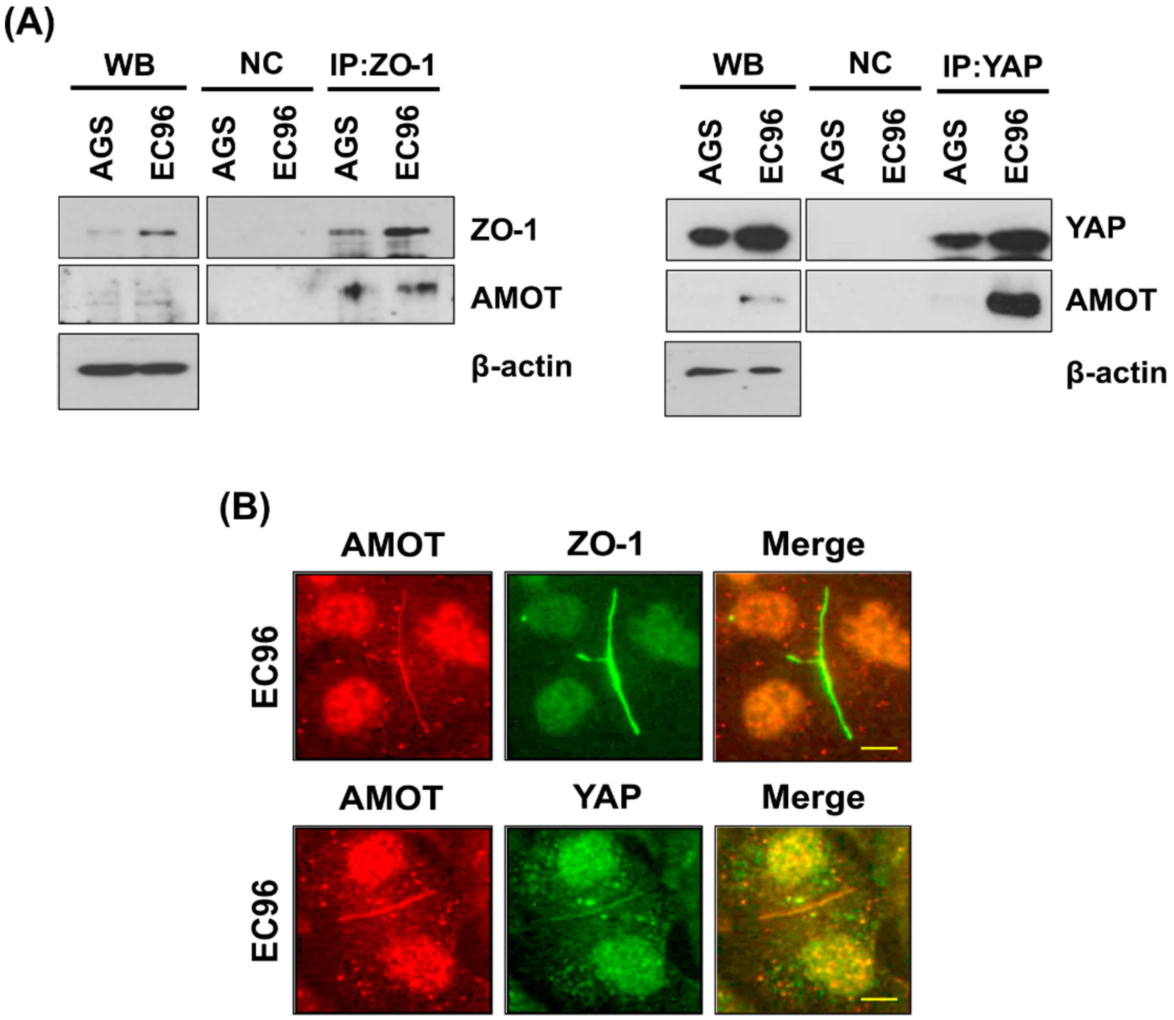

Fig. S4. AMOT is associated with YAP and ZO-1. (A) AGS and EC96 cells were subjected to IP analysis using anti-ZO-1 and anti-YAP antibodies and precipitates were subjected to immunoblot analysis for AMOT. (B) AGS and EC96 cells were subjected to IF analysis of AMOT to evaluate co-localization with ZO-1 or YAP. Scale bar = 10 μm.

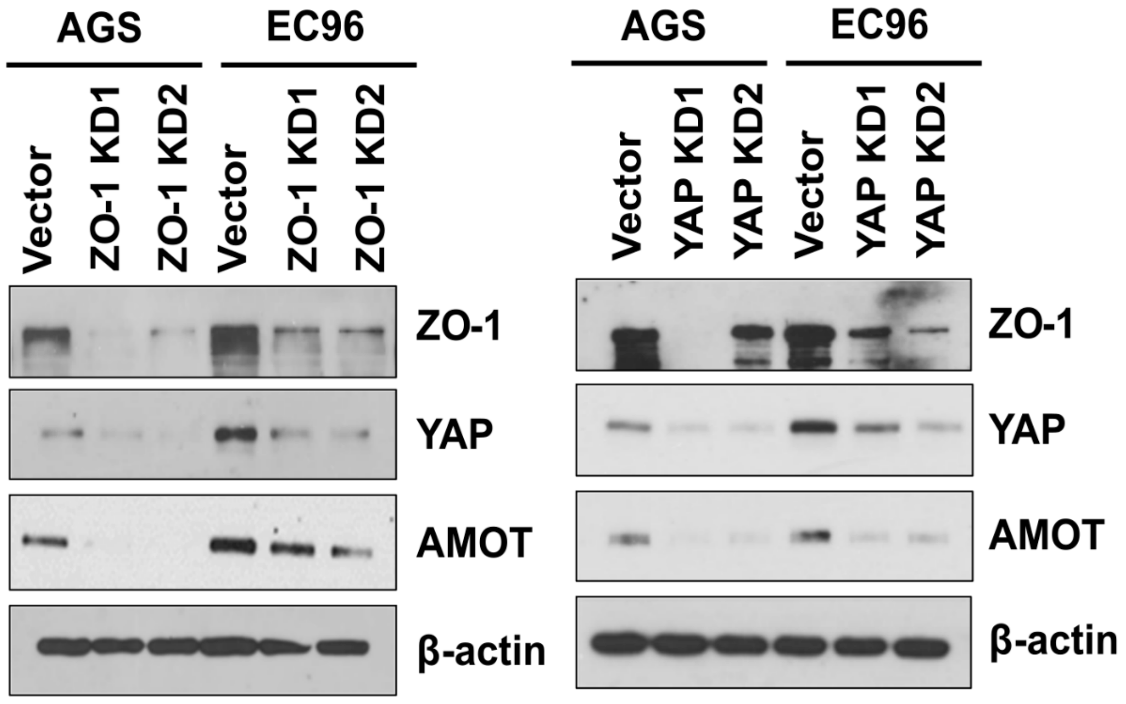

Fig. S5. Regulation of AMOT expression in ZO-1 KD or YAP KD cells. AGS and EC96 ZO-1 KD cells (left panel) or YAP KD cells (right panel) were subjected to immunoblot analysis using the indicated antibodies.

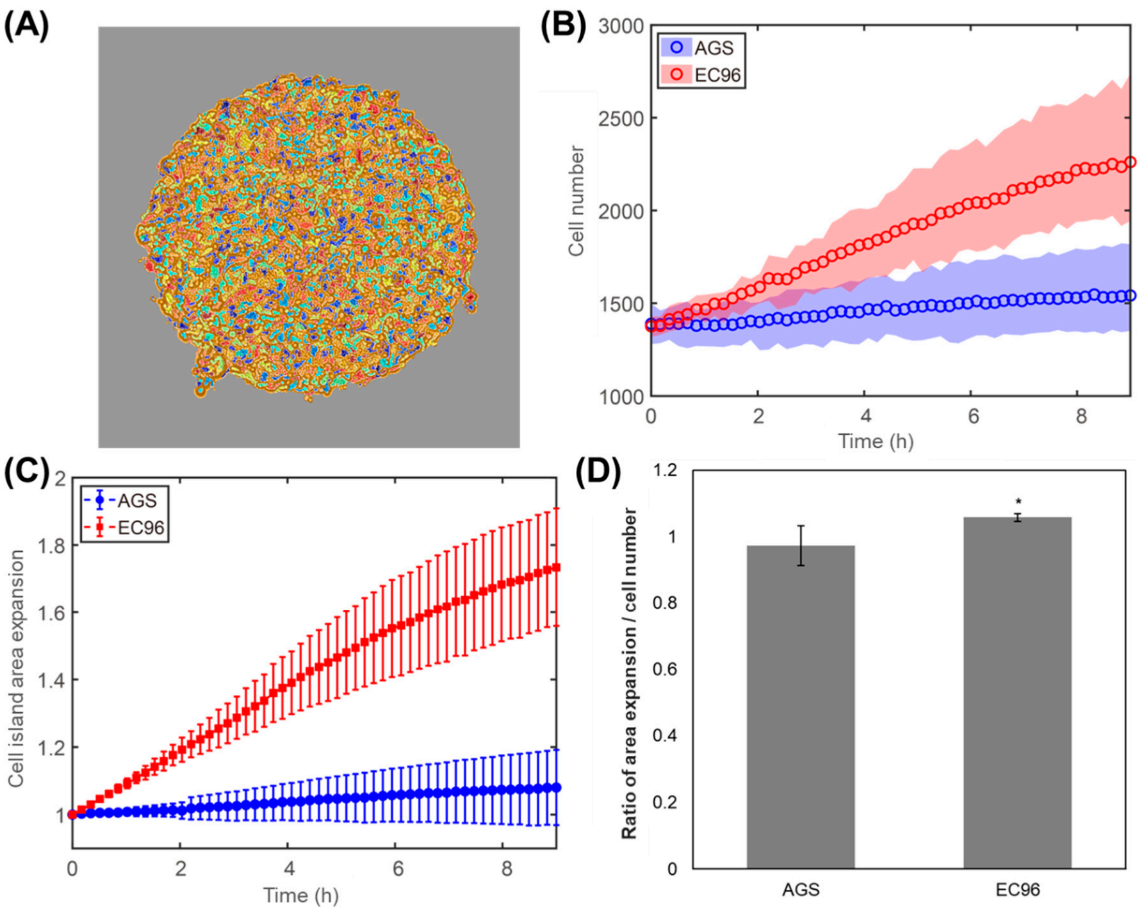

Fig. S6. Analysis of relationship between cell proliferation and cell island area expansion in AGS and EC96. (A) Each cell color label indicated the individual cells that were counted in a cell island. (B) Cell number was counted and plotted according to elapsed time after initial measurement to show increase of cell number in a time-dependent manner. Averages of five independent experiments are presented by circle and error bars are presented by color area. (C) Cell island area expansion was measured according to elapsed time. Averages of five independent experiments with error bars are presented. (D) Expanded cell island was divided by initial cell number and the ratio was presented. The error bars represent the standard errors calculated from separate five assays on each group. * $p < 0.05$
