# Supplementary material for: Yes-Associated Protein Is Required for ZO-1-Mediated Tight-Junction Integrity and Cell Migration in E-Cadherin-Restored AGS Gastric Cancer Cells
Source: Biomedicines. 2021 Sep 18;9(9):1264. doi: 10.3390/biomedicines9091264 (PMC8467433; doi:10.3390/biomedicines9091264)
Supplement: Supplementary file 1 [file biomedicines-09-01264-s001.zip › Table S1.pdf]

Table S1. The sequences of siRNAs		
Name		Sequences
Negative control	Forward	UUCUUCGAACGUGUCACGUTT
	Reverse	ACGUGACACGUUCGGAGAATT
YAP1	Forward	GACAUCUUCUGGUCAGAGATT
	Reverse	UCUCUGACCAGAAGAUGUCTT
YAP2	Forward	CUGCCACCAAGCUAGAUAATT
	Reverse	UUAUCUAGCUUGGUGGCAGTT
