# Supplementary material for: Yes-Associated Protein Is Required for ZO-1-Mediated Tight-Junction Integrity and Cell Migration in E-Cadherin-Restored AGS Gastric Cancer Cells
Source: Biomedicines. 2021 Sep 18;9(9):1264. doi: 10.3390/biomedicines9091264 (PMC8467433; doi:10.3390/biomedicines9091264)
Supplement: Supplementary file 1 [file biomedicines-09-01264-s001.zip › Table S2.pdf]

Table S2. Primer sequences for qRT-PCR		
Gene name		Sequence
ZO-1	Forward	TGCCATTACACGGTCCTCTG
	Reverse	GGTTCTGCCTCATCATTTCCTC
YAP	Forward	GGATTTCTGCCTTCCCTGAA
	Reverse	GATAGCAG GCGTGAGGAAC
CTGF	Forward	ACCGACTGGAAGACACGTTTG
	Reverse	CCAGGTCAGCTTCGCAAGG
Cyr61	Forward	GGTCAAAGTTACCGGGCAGT
	Reverse	GGAGGCATCGAATCCCAGC
β-actin	Forward	ATCTACGAGGGGTATGCC
	Reverse	TAGCTCTTCTCCAGGGAG
